# Microstructure Evolution and Flow Stress Model of a 20Mn5 Hollow Steel Ingot during Hot Compression

**DOI:** 10.3390/ma11040463

**Published:** 2018-03-21

**Authors:** Min Liu, Qing-Xian Ma, Jian-Bin Luo

**Affiliations:** Department of Mechanical Engineering, Tsinghua University, Beijing 100084, China; liumin881120@126.com (M.L.); luojblqw@mail.tsinghua.edu.cn (J.-B.L.)

**Keywords:** 20Mn5 steel, microstructure evolution, hot compression, flow stress model

## Abstract

20Mn5 steel is widely used in the manufacture of heavy hydro-generator shaft due to its good performance of strength, toughness and wear resistance. However, the hot deformation and recrystallization behaviors of 20Mn5 steel compressed under high temperature were not studied. In this study, the hot compression experiments under temperatures of 850–1200 °C and strain rates of 0.01/s–1/s are conducted using Gleeble thermal and mechanical simulation machine. And the flow stress curves and microstructure after hot compression are obtained. Effects of temperature and strain rate on microstructure are analyzed. Based on the classical stress-dislocation relation and the kinetics of dynamic recrystallization, a two-stage constitutive model is developed to predict the flow stress of 20Mn5 steel. Comparisons between experimental flow stress and predicted flow stress show that the predicted flow stress values are in good agreement with the experimental flow stress values, which indicates that the proposed constitutive model is reliable and can be used for numerical simulation of hot forging of 20Mn5 hollow steel ingot.

## 1. Introduction

Heavy cylinder forgings are widely used in key equipments such as nuclear pressure vessel, hydro-generator shaft and hydrogenation reactor [1].

Compared with solid steel ingot which is used for manufacture of cylinder forging, the advantages of adopting hollow steel ingot are as follows: (1) avoiding punching process; (2) increasing the utilization ratio of the steel ingot significantly; (3) reducing segregation and improving the material homogeneity as a result of a small solidification section and a high solidification speed; (4) requiring lower forging force [2].

20Mn5 steel has been widely used in the manufacture of hydro-generator shaft due to its good balance of strength, toughness, wear resistance and weldability. However, for heavy 20Mn5 hollow steel ingot, coarse grains and shrinkage cavities exist. To save the cost of production, it is necessary to carry out numerical simulation to obtain reasonable forging process parameters to make sure that the coarse grains can be refined and shrinkage cavities can be eliminated. In order to carry out a successful simulation of hot forging process, a precise constitutive model which describes the effect of temperature, strain rate and strain on stress is essential. At present, there is no report on the constitutive model of heavy 20Mn5 hollow steel ingot.

In this study, high temperature compression experiments of 20Mn5 steel under temperatures of 850–1200 °C and strain rates of 1/s, 0.1/s and 0.01/s are carried out using Gleeble thermal and mechanical simulation machine. Flow stress curves and microstructure after hot compression on different temperatures and strain rates are obtained. Based on the experimental flow stress curves, a two-stage constitutive model is established by introducing the classical stress-dislocation relation and the kinetic equation of dynamic recrystallization(DRX) [3,4,5,6,7,8,9,10,11,12,13,14,15,16,17,18,19,20,21,22,23,24,25]. By comparisons of the predicted flow stress values and experimental flow stress values, the proposed physically-based constitutive model shows high accuracy. Therefore, the newly developed constitutive model of 20Mn5 steel can be used for numerical simulation of hot forging process. Moreover, the flow stress curves and microstructure after hot compression can provide important reference for establishment of forging process specification of 20Mn5 heavy hydro-generator shaft.

## 2. Materials and Methods

### 2.1. Experimental Material

**Figure 1:**
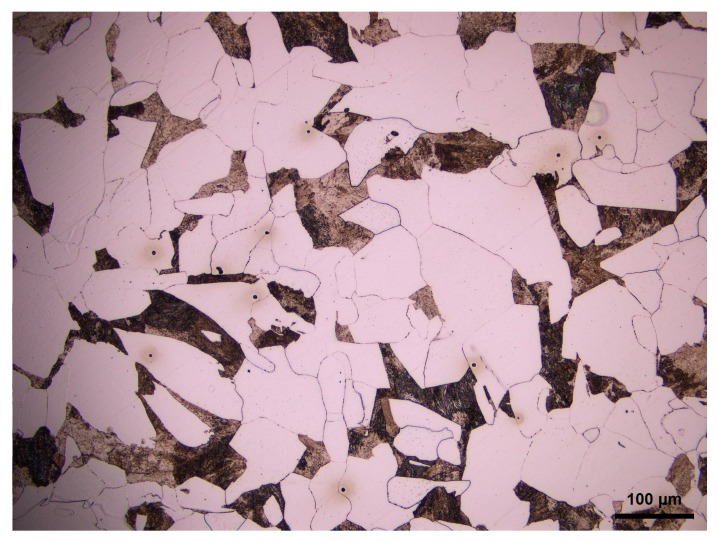
The initial as-cast microstructure of 20Mn5 steel.

The cylindrical samples with the diameter of 8 mm and the height of 12mm were taken from a 20Mn5 hollow steel ingot used for heavy hydro-generator shaft. The chemical composition of 20Mn5 steel is given in Table 1. Figure 1 shows the initial as-cast microstructure of 20Mn5 steel, which contains ferrite and pearlite.

### 2.2. Experimental Procedure

The hot compression experiments were carried out on a Gleeble-1500D thermal and mechanical simulation machine (DSI, NY, USA). Firstly, the specimens were heated to 1000 °C at a heating rate of 5 °C/s and held for 3 min. Then, the temperature was adjusted to deformation temperature (850–1200 °C with a 50 °C interval) at 10 °C/s and held for 60 s to get a uniform temperature distribution. Then, the specimens were compressed to a true strain of 0.7 under deformation temperatures of 850–1200 °C with a 50 °C interval and strain rates of 1/s, 0.1/s and 0.01/s. Finally, the specimens were quenched with water in order to retain the morphologies of austenite grains. After the hot compression experiments, the specimens were cut, ground, polished and etched in the mixture of 5 g picric acid, 4 g sodium dodecyl benzene sulfonate and 100 ml water at 60–70 °C. Then, the morphologies of emerged austenite grains were observed using an OLYMPUS BX51 microscope (Olympus, Tokyo, Japan).

## 3. Results and Discussion

### 3.1. Flow Stress Curves

**Figure 2:**
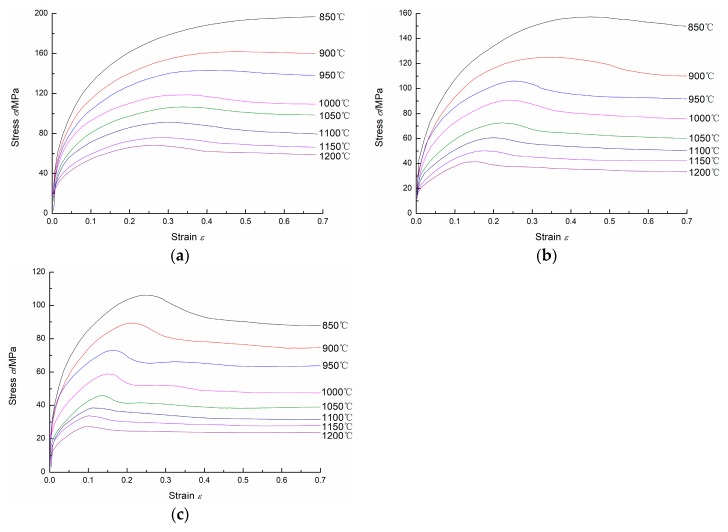
Flow stress curves of the 20Mn5 steel under strain rates of: (**a**) 1/s; (**b**) 0.1/s; (**c**) 0.01/s.

Figure 2 shows the flow stress curves of 20Mn5 steel compressed at different temperatures and different strain rates. As shown in Figure 2, for the same strain rate and strain, the flow stress is larger when the specimen is compressed at lower temperature; for the same temperature and strain, the flow stress is larger when the specimen is compressed at higher strain rate. And it can be easily seen that at high temperatures and low strain rates, the flow stress increases to a peak stress at first, and then reduces to a steady state stress and remains as a constant as the strain increases. For low temperatures and high strain rates, the steady state stress doesn’t occur. Moreover, for the same strain rate, the peak strain and steady state strain increase when the compression temperature decreases; for the same temperature, the peak strain and steady state strain increase when the strain rate increases.

For 20Mn5 steel, the flow stress is influenced by work hardening(WH), dynamic recovery(DRV) and dynamic recrystallization(DRX), which compete during hot compression. In the initial deformation stage, working hardening and dynamic recovery occur. Dislocation movement and rearrangement can reduce the dislocation density, but working hardening comes into prominence. As a result, the flow stress increases as the strain increases. When strain reaches a critical value, dynamic recrystallization occurs and the softening effect caused by dynamic recrystallization dominates. Correspondingly, the flow stress increases gradually until a peak stress at first, and then decreases as the strain increases. Finally, a dynamic balance between working hardening, dynamic recovery and dynamic recrystallization is reached. And the flow stress remains to be a constant as the strain increases.

### 3.2. Effect of Temperature and Strain Rate on Microstructure after Hot Compression

**Figure 3:**
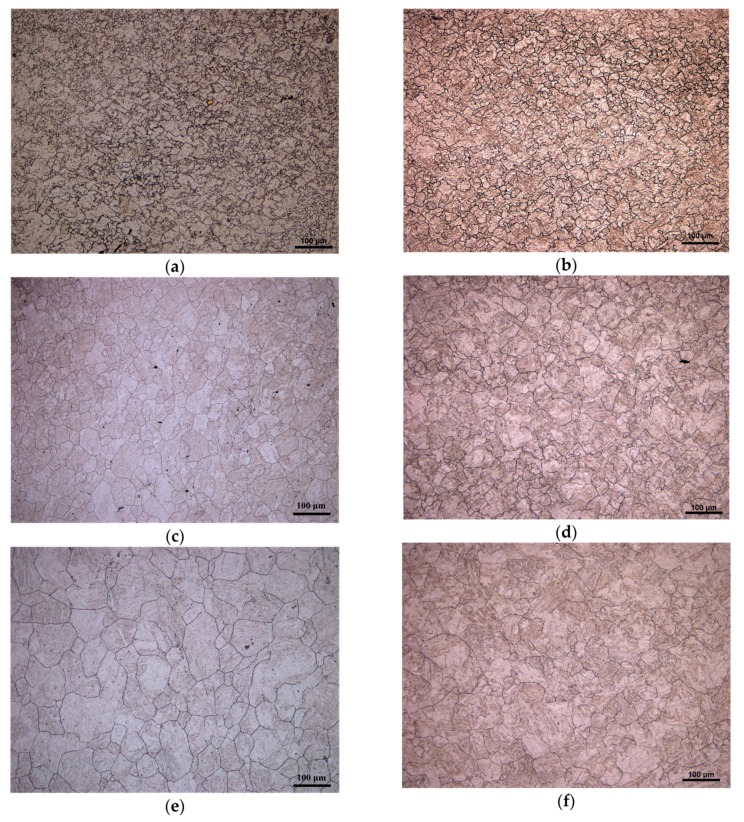

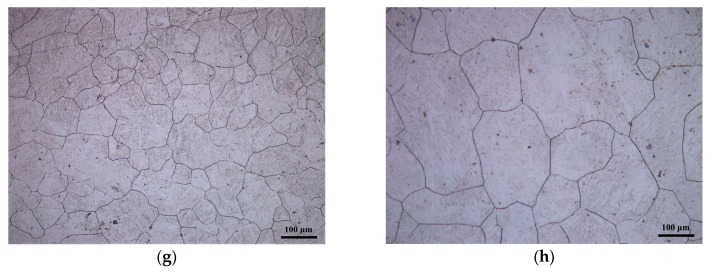
Microstructure of 20Mn5 steel compressed to a true strain of 0.7 under strain rate of 0.01/s and temperatures of: (**a**) 850 °C; (**b**) 900 °C; (**c**) 950 °C; (**d**) 1000 °C; (**e**) 1050 °C; (**f**) 1100 °C; (**g**) 1150 °C; (**h**) 1200 °C.

Figure 3 shows the microstructure of 20Mn5 steel compressed to a true strain of 0.7 under strain rate of 0.01 /s and different temperatures. It is obvious that the austenite grain size increases with the increase of the compression temperature. Under eight temperatures and strain rate of 0.01 /s, dynamic recrystallization occurs, respectively. The occurrence of dynamic recrystallization shown in Figure 3 can also be seen from the measured flow stress curves. In the above flow stress curves corresponding to Figure 3, the flow stress curves all reach a steady state when strain equals a certain value, which indicates that dynamic recrystallization is complete.

**Figure 4:**
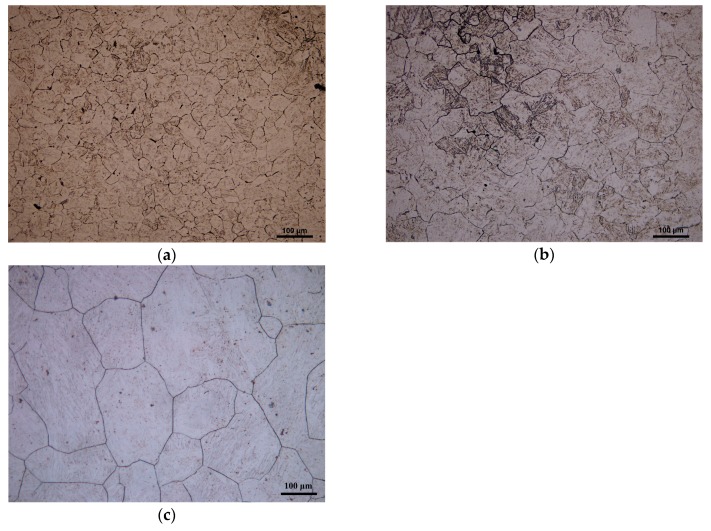
Microstructure of 20Mn5 steel compressed to a true strain of 0.7 under temperature of 1200 °C and strain rates of: (**a**) 1/s; (**b**) 0.1/s; (**c**) 0.01/s.

Figure 4 shows the microstructure of 20Mn5 steel compressed to a true strain of 0.7 under 1200 °C and different strain rates. It can be easily found that the higher the strain rate, the smaller the grain size. The reason for this phenomenon is high strain rate reduces the deformation time at a certain strain and the recrystallized nucleus has insufficient time to fully grow.

## 4. Establishment of A Two-Stage Constitutive Model and Its Verification

### 4.1. The Derivation of A Two-Stage Constitutive Model

**Figure 5:**
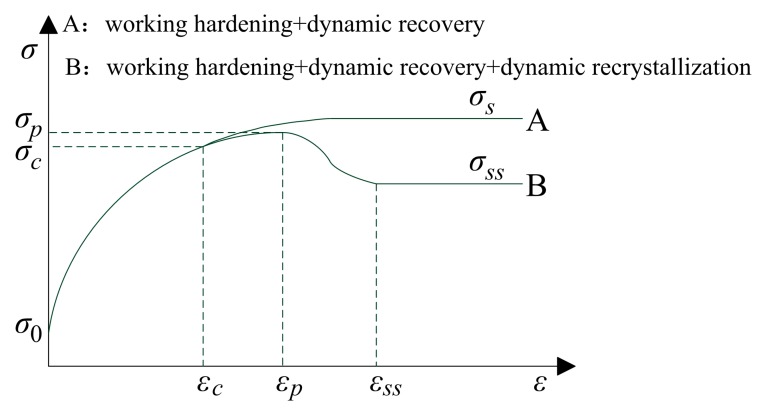
Typical flow stress curves for hot working.

Figure 5 shows two typical flow stress curves for hot working. In curve B, in the initial deformation stage (0 ≤ *ε* < *ε_c_*), stress increases from σ_0_ to *σ_c_* as a result of working hardening and dynamic recovery. When strain equals a critical value *ε_c_*, dynamic recrystallization occurs. And as strain increases, firstly, the stress increases from *σ_c_* to *σ_p_*, and then stress decreases form *σ_p_* to a steady state stress *σ_ss_*. If dynamic recrystallization never occurs in some materials, the change of stress with strain will be similar to curve A.

The flow stress curve of 20Mn5 steel in this study is similar to curve B, and a two-stage method is used to establish the flow stress model of 20Mn5 steel.

(1) stage of working hardening and dynamic recovery (0 ≤ *ε* < *ε_c_*)

The evolution of the dislocation density with strain is generally considered to be as follows:

(1) $$\frac{d\rho }{d\varepsilon }=k_{1}\sqrt{\rho }-k_{2}\rho ,$$

where *k*_1_ represents the coefficient of working hardening, and k_2_ is the coefficient of dynamic recovery.

When ε=0, $\rho =\rho_{0}$, where $\rho_{0}$ is the initial dislocation density.

By integration of Equation (1), dislocation density ρ can be expressed as follows:

(2) $$\rho =\left(\frac{k_{1}}{k_{2}}-\frac{k_{1}}{k_{2}}e^{-\frac{k_{2}}{2}\varepsilon }+\sqrt{\rho_{0}}e^{-\frac{k_{2}}{2}\varepsilon }\right){}^{2},$$

When $\frac{d\rho }{d\varepsilon }=0$, $\rho_{s}={\left(\frac{k_{1}}{k_{2}}\right)}^{2}$, where $\rho_{s}$ is the saturation dislocation density corresponding to the saturation stress $\sigma_{s}$.

Based on the above equations and the classical Taylor relation $\sigma =\alpha \mu b\sqrt{\rho }$, where α is the material constant, μ is the shear modulus and b is Burgers vector, the stress $\sigma_{WH}$ at strain ε can be expressed as follows:

(3) $$\sigma_{WH}=\sigma_{s}+(\sigma_{0}-\sigma_{s})e^{-\frac{k_{2}}{2}\varepsilon },\;(\varepsilon <\varepsilon_{c}),$$

(2) stage of dynamic recrystallization (*ε* ≥ *ε_c_*)

The volume fraction of DRX, $X_{drx}$, can be determined by the following equation:

(4) $$X_{drx}=1-\exp(-k_{d}{\left(\frac{\varepsilon -\varepsilon_{c}}{\varepsilon_{p}}\right)}^{n_{d}}),\;(\varepsilon \geq \varepsilon_{c})$$

where $X_{drx}$ is the volume fraction of DRX, $\varepsilon_{c}$ is the critical strain, $\varepsilon_{p}$ is the peak strain, $k_{d}$ and $n_{d}$ are constants related to material.

The relation between $X_{drx}$ and stress parameters can be given as follows:

(5) $$X_{drx}=\frac{\sigma_{WH}-\sigma }{\sigma_{s}-\sigma_{ss}},\;(\varepsilon \geq \varepsilon_{c})$$

where $\sigma_{s}$ is the saturation stress, $\sigma_{ss}$ is the steady state stress, $\sigma_{WH}$ is the stress at strain ε calculated by Equation (3), and σ is flow stress at strain ε.

By combining Equations (4) and (5), the flow stress during DRX period can be given by the following expression:

(6) $$\sigma =\sigma_{WH}-(\sigma_{s}-\sigma_{ss})(1-\exp(-k_{d}{\left(\frac{\varepsilon -\varepsilon_{c}}{\varepsilon_{p}}\right)}^{n_{d}})),\;(\varepsilon \geq \varepsilon_{c})$$

### 4.2. Determination of Material Constants(α, n, Q and A) Based on the Peak Stress

The Arrhenius equation proposed by Sellars and Tegart is widely used to describe the relationship between flow stress, strain rate and temperature:

(7) $$\dot{\varepsilon }=A{\left[\sinh(\alpha \sigma )\right]}^{n}\exp(-\frac{Q}{RT}),$$

where A, α and n are material constants; Q is the activation energy of deformation (J/mol); $\dot{\varepsilon }$ is strain rate (s^−1^); σ is flow stress (MPa); T is the absolute temperature (K); and R is the gas constant (8.314 J/(mol·K)).

Based on Taylor expansion, the following equations can be obtained:

(8) $$e^{\alpha \sigma }=1+\alpha \sigma +\frac{{\left(\alpha \sigma \right)}^{2}}{2!}+\frac{{\left(\alpha \sigma \right)}^{3}}{3!}+\cdots ,$$

(9) $$e^{-\alpha \sigma }=1+(-\alpha \sigma )+\frac{{\left(-\alpha \sigma \right)}^{2}}{2!}+\frac{{\left(-\alpha \sigma \right)}^{3}}{3!}+\cdots ,$$

(10) $$\sinh(\alpha \sigma )=\frac{e^{\alpha \sigma }-e^{-\alpha \sigma }}{2}=\alpha \sigma +\frac{{\left(\alpha \sigma \right)}^{3}}{3!}+\cdots ,$$

According to Equations (7) and (10), the following equations can be easily obtained:

(11) $$\dot{\varepsilon }=A_{1}\sigma^{n_{1}}\exp(-\frac{Q}{RT}),\;\mathrm{for}\;\mathrm{low}\;\mathrm{stress}\;\mathrm{level}$$

(12) $$\dot{\varepsilon }=A_{2}\exp(\beta \sigma )\exp(-\frac{Q}{RT}),\;\mathrm{for}\;\mathrm{high}\;\mathrm{stress}\;\mathrm{level}$$

According to Equations (7), (11) and (12), the following equation is obvious:

(13) $$\alpha =\frac{\beta }{n_{1}},$$

Taking the natural logarithms on both sides of Equations (7), (11) and (12), the following expressions can be obtained:

(14) $$\ln\dot{\varepsilon }=\ln A+n\ln[\sinh(\alpha \sigma )]+(-\frac{Q}{RT}),$$

(15) $$\ln\dot{\varepsilon }=\ln A_{1}+(-\frac{Q}{RT})+n_{1}\ln\sigma ,$$

(16) $$\ln\dot{\varepsilon }=\ln A_{2}+(-\frac{Q}{RT})+\beta \sigma ,$$

**Figure 6:**
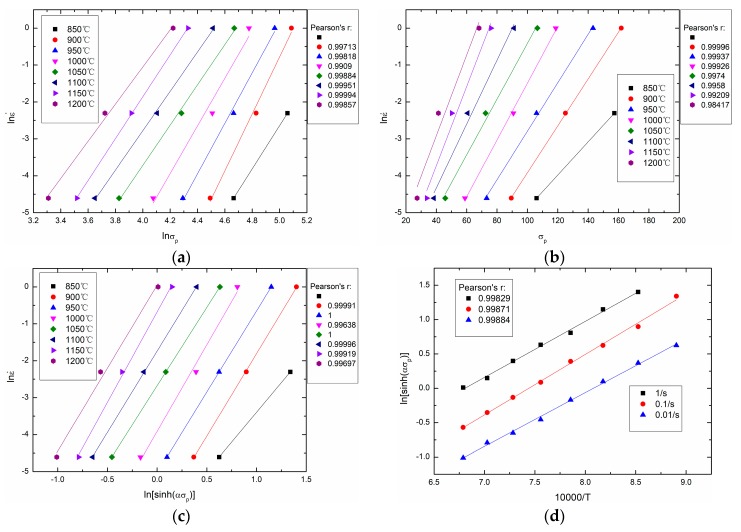
Calculation of material constants: (**a**) *n_1_*; (**b**) β; (**c**) *n*; (**d**) Q and *A*.

In this study, σ in the above equations is taken as the peak stress $\sigma_{p}$. Based on experimental data and Equations (14)–(16), Figure 6 is drawn as follows. In Figure 6a, the slopes of the lines equal *n*_1_, and *n*_1_ = 6.040465 is obtained. In Figure 6b, the slopes of the lines equal *β*, and *β* = 0.078723 is obtained. Thus, *α* = *β*/*n*_1_ = 0.013033. In Figure 6c, the slopes of the lines equal *n*, and *n* = 4.346973 is obtained. In Figure 6d, the slopes of the lines equal *Q*/(10000*nR*), and the intercepts of the lines equal −(1/*n*)ln*A* + (1/*n*)ln $\dot{\varepsilon }$. Thus, *Q* = 298571.8169 J/mol and *A* = 8.85 × 10^10^ can be obtained.

From Equation (7), the following equation can be obtained:

(17) $$\sigma_{p}=\frac{\ln({\left(\frac{Z}{A}\right)}^{\frac{1}{n}}+\sqrt{{\left(\frac{Z}{A}\right)}^{\frac{2}{n}}+1})}{\alpha }=\frac{\ln({\left(\frac{\overset{\cdot }{\varepsilon }\exp(\frac{Q}{RT})}{A}\right)}^{\frac{1}{n}}+\sqrt{{\left(\frac{\overset{\cdot }{\varepsilon }\exp(\frac{Q}{RT})}{A}\right)}^{\frac{2}{n}}+1})}{\alpha },$$

where $Z=\overset{\cdot }{\varepsilon }\exp(\frac{Q}{RT})$, Z is Zener-Hollomon parameter, and Z represents the strain rate compensated by temperature.

Based on the calculated material constants (α, n, Q and A), the peak stress $\sigma_{p}$ at any temperature T and any strain rate $\dot{\varepsilon }$ can be obtained.

### 4.3. Determination of Material Parameters(ε_p_, ε_c_, σ_s_, σ_ss_, σ_0_, k_2_, k_d_, n_d_) in the Two-Stage Constitutive Model

The peak strain $\varepsilon_{p}$ can be obtained directly from the experimental flow stress curves. The mathematical model of $\varepsilon_{p}$ can be expressed as follows:

(18) $$\varepsilon_{p}=kZ^{m},$$

**Figure 7:**
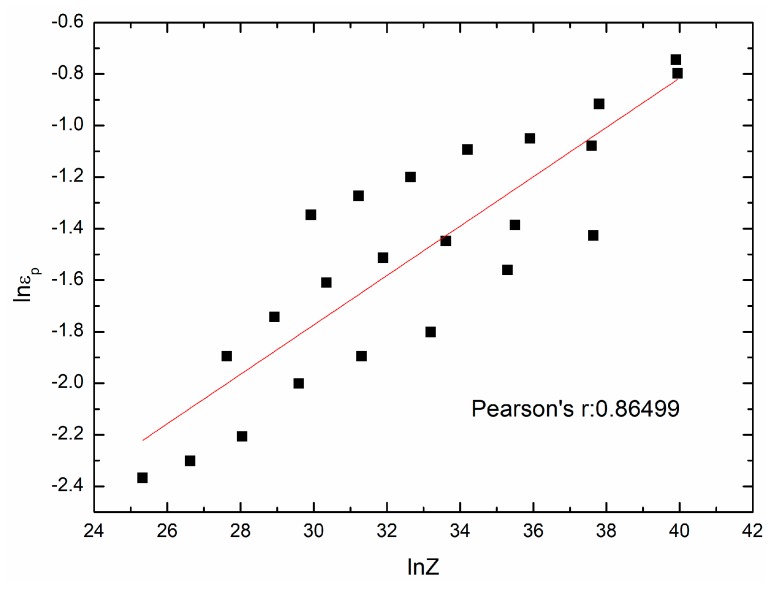
The relationship between $\ln\varepsilon_{p}$ and lnZ.

By taking natural logarithm on both sides of Equation (18) and using different groups of $\varepsilon_{p}$, $\dot{\varepsilon }$, T and Q, Figure 7 can be drawn as follows. According to the fit result in Figure 7, $\varepsilon_{p}$ can be expressed as a function of Z:

(19) $$\varepsilon_{p}=0.0095812Z^{0.095811},$$

The critical strain and critical stress can be obtained from θ-σ (working hardening rate θ=dσ/dε) curve. KIM et al. proposes that θ-σ curve can be divide into three segments [26,27]. The first segment starts from $\sigma_{0}$ and ends with $\sigma_{c}$, during which working hardening and dynamic recovery exist, and working hardening rate is positive. The second segment starts from $\sigma_{c}$ and ends with $\sigma_{p}$, during which dynamic recrystallization exists and working hardening rate is also positive. The third segment starts from $\sigma_{p}$ and ends with $\sigma_{ss}$, during which working hardening rate is negative. Drawing a tangent line at the critical point ($\sigma_{c}$,$\theta_{c}$), the intersection of the tangent line and σ axis is ($\sigma_{s}$, 0).

**Figure 8:**
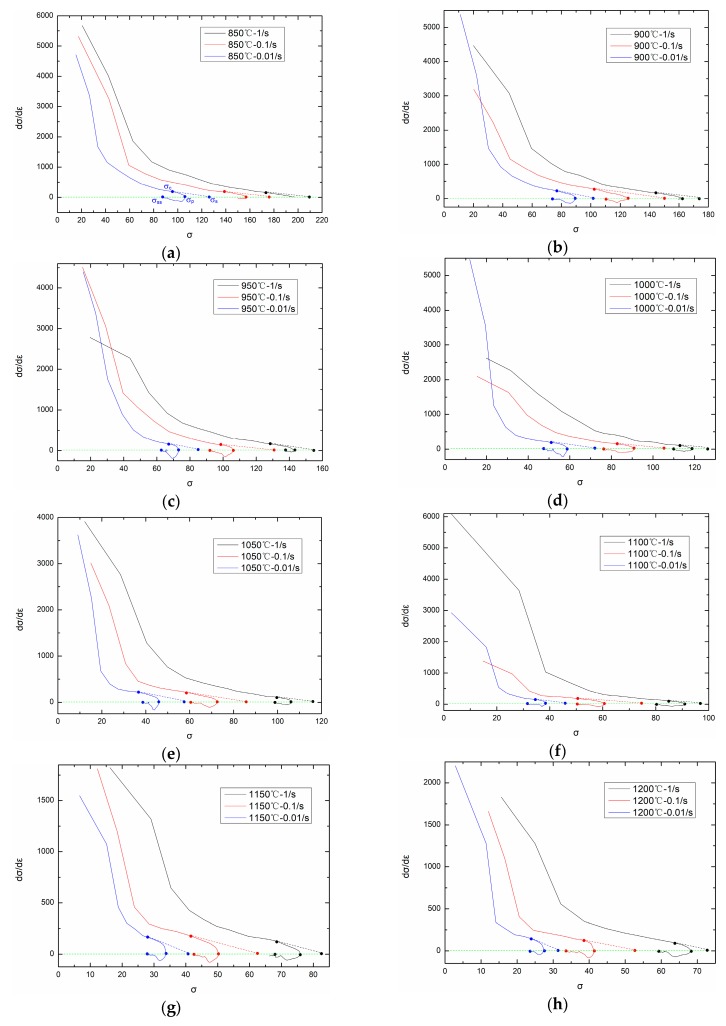
dσ/dε-σ curves on different temperatures: (**a**) 850 °C; (**b**) 900 °C; (**c**) 950 °C; (**d**) 1000 °C; (**e**) 1050 °C; (**f**) 1100 °C; (**g**) 1150 °C; (**h**) 1200 °C.

Figure 8 shows the θ-σ curves under different temperatures and strain rates. From Figure 8, critical stress $\sigma_{c}$ on different temperatures and strain rates can be obtained (The second derivative $d^{2}\theta /d\sigma^{2}=0$ when $\sigma =\sigma_{c}$). Based on experimental flow stress curves, the critical strain $\varepsilon_{c}$ corresponding to critical stress $\sigma_{c}$ can be easily obtained. By comparing critical strain $\varepsilon_{c}$ and peak strain $\varepsilon_{p}$ on each temperature and strain rate, the following relationship can be obtained:

(20) $$\varepsilon_{c}=0.83\varepsilon_{p},$$

The saturation stress $\sigma_{s}$ can be obtained by the tangent line method mentioned above. The steady state stress $\sigma_{ss}$ can be obtained from the experimental flow stress curves. The mathematical models of $\sigma_{s}$ and $\sigma_{ss}$ can be expressed as follows:

(21) $$\sinh(\alpha \sigma_{s})=kZ^{m},$$

(22) $$\sinh(\alpha \sigma_{ss})=kZ^{m},$$

**Figure 9:**
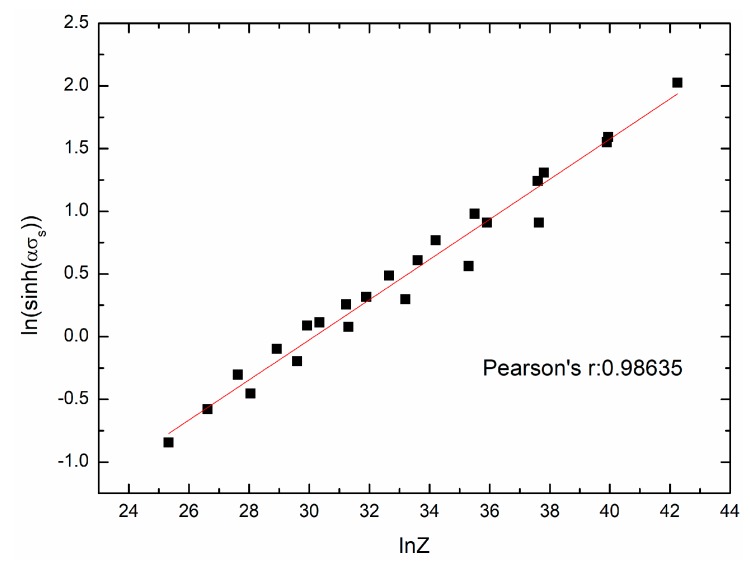
The relationship between $\ln[\sinh(\alpha \sigma_{s})]$ and lnZ.

**Figure 10:**
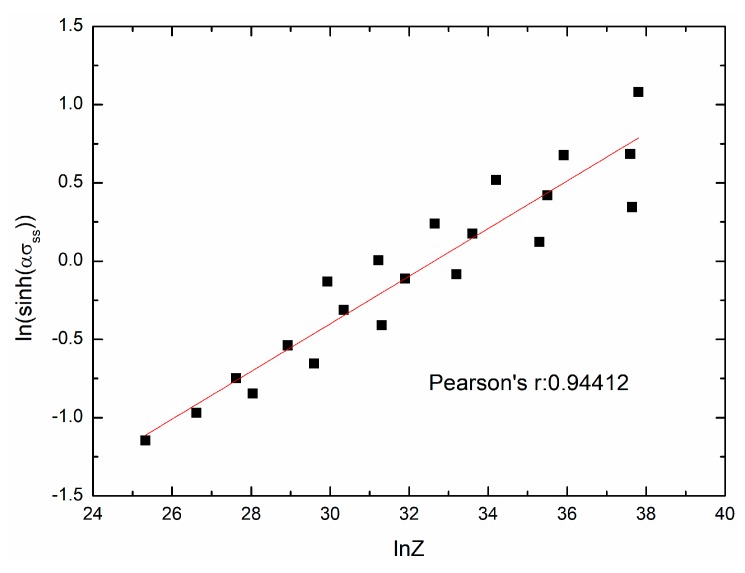
The relationship between $\ln[\sinh(\alpha \sigma_{ss})]$ and lnZ.

Figure 9 and Figure 10 show the fit results of $\ln[\sinh(\alpha \sigma_{s})]-\ln Z$ and $\ln[\sinh(\alpha \sigma_{ss})]-\ln Z$, respectively. The mathematical models can be given as:

(23) $$\sinh(0.013033\sigma_{s})=0.0080007Z^{0.160114},$$

(24) $$\sinh(0.013033\sigma_{ss})=0.0069625Z^{0.152207},$$

**Figure 11:**
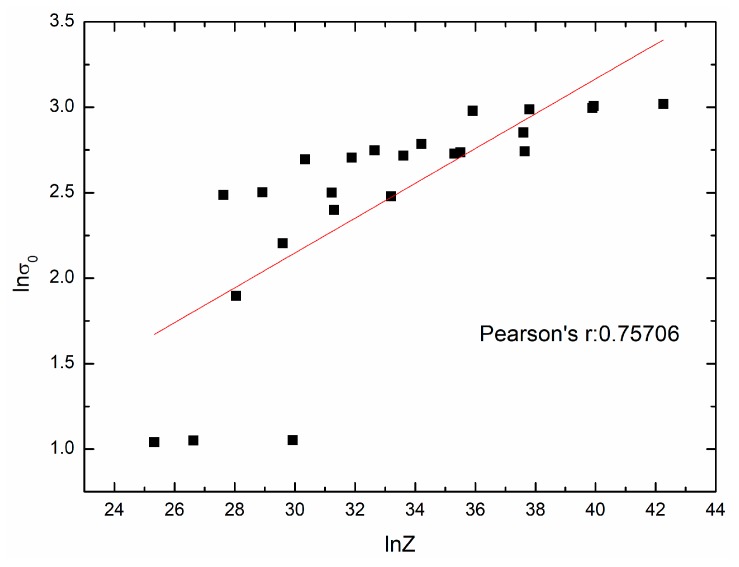
The relationship between $\ln\sigma_{0}$ and lnZ.

The mathematical model of initial stress $\sigma_{0}$ can also be obtained with the method mentioned above. Figure 11 shows the fit result. And the following expression can be given:

(25) $$\sigma_{0}=0.40403Z^{0.10180},$$

The constant $k_{2}$ on different temperatures and strain rates can be given by the following expression:

(26) $$k_{2}=\ln\frac{\sigma -\sigma_{s}}{\sigma_{0}-\sigma_{s}}(-2)/\varepsilon ,$$

**Figure 12:**
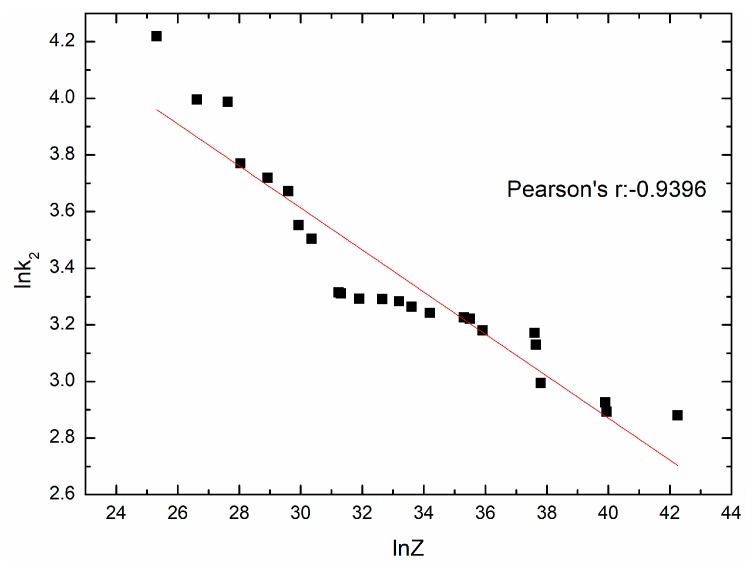
The relationship between $\ln k_{2}$ and lnZ.

Based on the stress-strain data ($\varepsilon <\varepsilon_{c}$), many values of $k_{2}$ on each temperature and strain rate can be obtained, and the average value is taken. Figure 12 shows the fit result. And the mathematical model is as follows:

(27) $$k_{2}=343.60152Z^{-0.074220},$$

The volume fraction of DRX can be given as follows:

(28) $$X_{drx}=\frac{\sigma_{WH}-\sigma }{\sigma_{s}-\sigma_{ss}},\;(\varepsilon \geq \varepsilon_{c})$$

**Figure 13:**
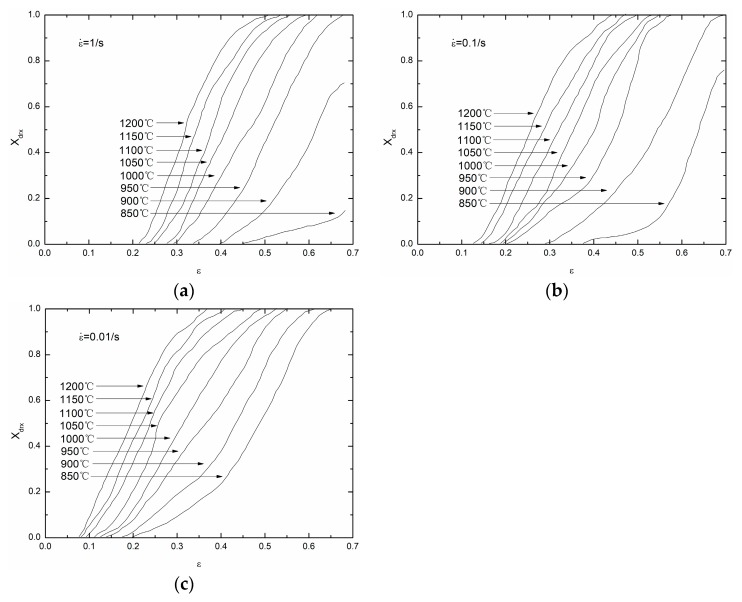
The $X_{drx}$-ε curves on different temperatures and strain rates: (**a**) 1/s; (**b**) 0.1/s; (**c**) 0.01/s.

Based on Equation (28), different groups of ($X_{drx}$, ε) on each temperature and strain rate can be obtained. Figure 13 shows the $X_{drx}$-ε curves on different temperatures and strain rates.

Making some transformations and taking natural logarithm on the kinetic model of DRX can give:

(29) $$\ln(-\ln(1-X_{drx}))=\ln k_{d}+n_{d}\ln(\frac{\varepsilon -\varepsilon_{c}}{\varepsilon_{p}}),\;(\varepsilon \geq \varepsilon_{c})$$

**Figure 14:**
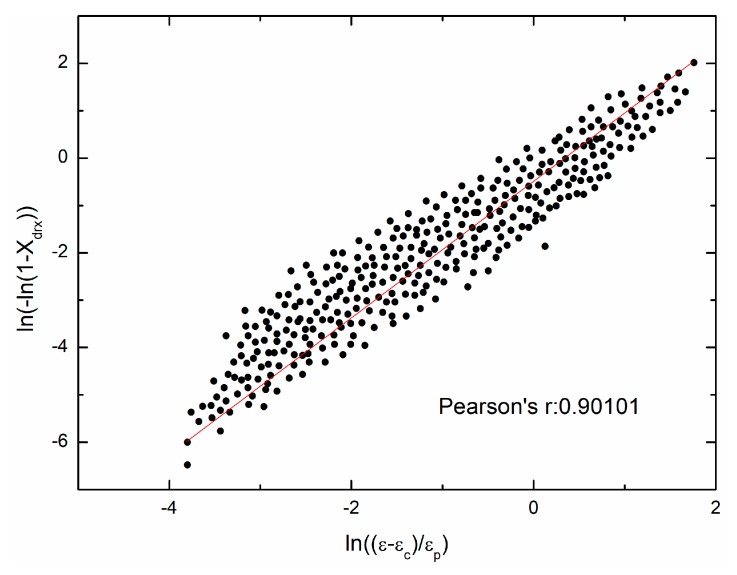
The relationship between $\ln(-\ln(1-X_{drx}))$ and $\ln(\frac{\varepsilon -\varepsilon_{c}}{\varepsilon_{p}})$.

Figure 14 shows the fit result. And $n_{d}$ = 1.4545, $k_{d}$ = 0.6065 are obtained.

### 4.4. Verification of the Proposed Two-Stage Constitutive Model

**Figure 15:**
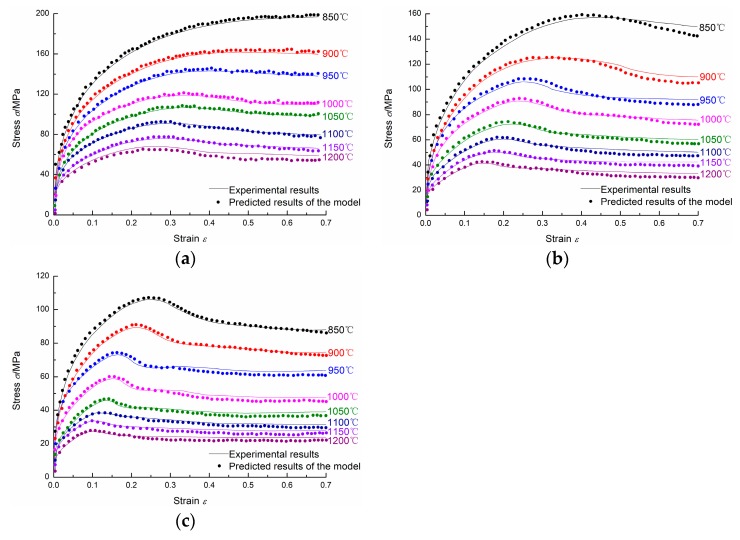
The comparison between experimental results and predicted results of the model: (**a**) 1/s; (**b**) 0.1/s; (**c**) 0.01/s.

Based on the activation energy *Q* and the material parameters ($\varepsilon_{p}$, $\varepsilon_{c}$, $\sigma_{s}$, $\sigma_{ss}$, $\sigma_{0}$, $k_{2}$, $n_{d}$, and $k_{d}$) determined above, the flow stress under any temperature, strain rate, and strain can be calculated. Figure 15 shows the comparison between experimental results and predicted results of the model. It can be easily seen that the predicted flow stress values are in good agreement with the experimental flow stress values. Thus, the proposed two-stage constitutive model can give a reasonable estimate of the flow stress of 20Mn5 steel and can be used for numerical simulation of hot forging of 20Mn5 hollow steel ingots.

## 5. Conclusions

(1) During hot compression of 20Mn5 steel, firstly, the flow stress increases to a peak stress, and then the flow stress decreases gradually to a steady state stress with the increase of strain. Peak stress does not occur only at 850 °C and 1/s. At higher temperatures and lower strain rates, the peak strain, steady state strain, peak stress, and steady state stress are smaller.
(2) The material constants (*α*, *n*, *Q*, and *A*) are calculated based on the peak stress.
(3) A two-stage constitutive model of 20Mn5 steel is established. The related material parameters (*ε_p_*, *ε_c_*, *σ_s_*, *σ_ss_*, *σ*_0_, *k*_2_, *n_d_*, and *k_d_*) are determined.
(4) The proposed constitutive model can give a good prediction of the flow stress for different temperatures, strain rates, and strains, and can be used for numerical simulation of hot forging of 20Mn5 hollow steel ingots.

## Figures and Tables

**Table 1:** Chemical composition of 20Mn5 steel (wt %).

C	Si	Mn	P	S	Cr	Ni	Mo	Al
0.24	0.26	1.47	0.0084	0.0020	0.15	0.076	0.020	0.015

## References

[1] Liu M., Ma Q.X. (2016). Research on rotatory deformation uniformity and compaction effect of super-heavy hollow steel ingot. J. Mech. Eng..

[2] Liu M., Dong X.L., Ma Q.X. (2013). Investigation on hollow steel ingot forging process of heavy cylinder forging. J. Plast. Eng..

[3] Mao W.M., Zhao X.B. (1994). Recrystallization and Grain Growth of Metal.

[4] Chen F., Feng G.W., Cui Z.S. (2016). New constitutive model for hot working. Metall. Mater. Trans. A..

[5] Dong D.Q., Chen F., Cui Z.S. (2015). A physically-based constitutive model for SA508-III steel: modeling and experimental verification. Mater. Sci. Eng. A..

[6] Chen F., Cui Z.S., Chen S.J. (2011). Recrystallization of 30Cr2Ni4MoV ultra-super-critical rotor steel during hot deformation. Part I: dynamic recrystallization. Mater. Sci. Eng. A..

[7] Chen F., Liu J., Ou H.G., Liu B., Cui Z.S., Long H. (2015). Flow characteristics and intrinsic workability of IN718 superalloy. Mater. Sci. Eng. A..

[8] Li X.C., Duan L.L., Li J.W., Wu X.C. (2015). Experimental study and numerical simulation of dynamic recrystallization behavior of a micro-alloyed plastic mold steel. Mater. Des..

[9] Wang J., Zhao G.Q., Chen L., Li J.L. (2016). A comparative study of several constitutive models for power metallurgy tungsten at elevated temperature. Mater. Des..

[10] Lin Y.C., Chen X.M. (2011). A critical review of experimental results and constitutive descriptions for metals and alloys in hot working. Mater. Des..

[11] Wang J., Yang H.T., Wang X.G., Xiao H. (2015). A new mathematical model for predicting flow stress up to the critical strain during hot deformation. Mater. Des..

[12] Solhjoo S., Vakis A.I., Pei Y.T.T. (2017). Two phenomenological models to predict the single peak flow stress curves up to the peak during hot deformation. Mech. Mater..

[13] Ji G.L., Li Q., Li L. (2014). A physical-based constitutive relation to predict flow stress for Cu-0.4Mg alloy during hot working. Mater. Sci. Eng. A.

[14] Ji G.L., Li Q., Ding K.Y., Yang L., Li L. (2015). A physical-based constitutive model for high temperature deformation of Cu-0.36Cr-0.03Zr alloy. J. Alloys Compd..

[15] Ji G.L., Li L., Qin F.L., Zhu L.Y., Li Q. (2017). Comparative study of phenomenological constitutive equations for an as-rolled M50NiL steel during hot deformation. J. Alloys Compd..

[16] Ji G.L., Li F.G., Li Q.H., Li H.Q., Li Z. (2010). Research on the dynamic recrystallization kinetics of Aermet100 steel. Mater. Sci. Eng. A..

[17] Ji G.L., Li Q., Li L. (2013). The kinetics of dynamic recrystallization of Cu-0.4Mg alloy. Mater. Sci. Eng. A.

[18] Busso E.P. (1998). A continuum theory for dynamic recrystallization with microstructure-related length scales. Int. J. Plast..

[19] McQueen H.J., Ryan N.D. (2002). Constitutive analysis in hot working. Mater. Sci. Eng. A..

[20] McQueen H.J. (2004). Development of dynamic recrystallization theory. Mater. Sci. Eng. A..

[21] McQueen H.J., Imbert C.A.C. (2004). Dynamic recrystallization: plasticity enhancing structural development. J. Alloys Compd..

[22] McQueen H.J., Yue S., Ryan N.D., Fry E. (1995). Hot working characteristics of steels in austenitic state. J. Mater. Process. Technol..

[23] Medina S.F., Hernandez C.A. (1996). Modelling of the dynamic recrystallization of austenite in low alloy and microalloyed steels. Acta Mater..

[24] Poliak E.I., Jonas J.J. (2007). Initiation of dynamic recrystallization in constant strain rate hot deformation. ISIJ Int..

[25] Stewart G.R., Jonas J.J., Montheillet F. (2004). Kinetics and critical conditions for the initiation of dynamic recrystallization in 304 stainless steel. ISIJ Int..

[26] Kim S.I., Yoo Y.C. (2001). Dynamic recrystallization behavior of AISI 304 stainless steel. Mater. Sci. Eng. A..

[27] Kim S.I., Lee Y., Lee D.L., Yoo Y.C. (2003). Modeling of AGS and recrystallized fraction of microalloyed medium carbon steel during hot deformation. Mater. Sci. Eng. A..

